# Biomarkers of Neurodegeneration in Autoimmune-Mediated Encephalitis

**DOI:** 10.3389/fneur.2018.00668

**Published:** 2018-09-19

**Authors:** Peter Körtvelyessy, Harald Prüss, Lorenz Thurner, Walter Maetzler, Deborah Vittore-Welliong, Jörg Schultze-Amberger, Hans-Jochen Heinze, Dirk Reinhold, Frank Leypoldt, Stephan Schreiber, Daniel Bittner

**Affiliations:** ^1^Department of Neurology, University Hospital Magdeburg, Magdeburg, Germany; ^2^German Center for Neurodegenerative Diseases Magdeburg, Magdeburg, Germany; ^3^Department of Neurology, Charité-Universitätsmedizin Berlin, Berlin, Germany; ^4^German Center for Neurodegenerative Diseases Berlin, Berlin, Germany; ^5^José Carreras Center for Immuno- and Gene Therapy and Internal Medicine I, Saarland University Medical School, Homburg, Germany; ^6^Department of Neurodegeneration, Hertie Institute for Clinical Brain Research (HIH), University of Tübingen, Tübingen, Germany; ^7^German Center for Neurodegenerative Diseases Tübingen, Tübingen, Germany; ^8^Department of Neurology, University Hospital Schleswig-Holstein, Kiel, Germany; ^9^Department of Neurology and Epileptology, Universitätsklinikum Tübingen, Universität Tübingen, Tübingen, Germany; ^10^Department of Neurology, Median Clinic Kladow, Kladow, Germany; ^11^Department of Behavioral Neurology, Leibniz Institute for Neurobiology, Magdeburg, Germany; ^12^Department of Immunohistopathology, Institute of Molecular and Clinical Immunology, Magdeburg, Germany; ^13^Asklepios Department of Neurology, Brandenburg a.d. Havel, Germany

**Keywords:** progranulin, neurofilament light chain, NMDAR encephalitis, Lgi-1 encephalitis, Caspr2 encephalitis, tau, autoimmune encephalitis

## Abstract

Progranulin (PGRN), Total-Tau (t-tau), and Neurofilament light chain (NfL) are well known biomarkers of neurodegeneration. The objective of the present study was to investigate whether these parameters represent also biomarkers in autoimmune-mediated Encephalitis (AE) and may give us insights into the pathomechanisms of AE. We retrospectively examined the concentration of PGRN in the cerebrospinal fluid (CSF) and serum of 38 patients suffering from AE in acute phase and/or under treatment. This AE cohort comprises patients with autoantibodies against: NMDAR (*n* = 18 patients), Caspr2 (*n* = 8), Lgi-1 (*n* = 10), GABAB(R) (*n* = 1), and AMPAR (*n* = 1). Additionally, the concentrations of NfL (*n* = 25) and t-tau (*n* = 13) in CSF were measured when possible. Follow up data including MRI were available in 13 patients. Several age-matched cohorts with neurological diseases besides neuroinflammation or neurodegeneration served as control groups. We observed that PGRN was significantly elevated in the CSF of patients with NMDAR-AE in the acute phase, but normalized at follow up under treatment (*p* < 0.01). In the CSF of other patients with AE PGRN was in the range of the CSF levels of control groups. T-tau was highly elevated in the CSF of patients with temporal FLAIR-signal in the MRI and in patients developing a hippocampal sclerosis. NfL was exceptionally high initially in Patients with AE with a paraneoplastic or parainfectious cause and also normalized under treatment. The normalizations of all biomarkers were mirrored in an improvement on the modified Rankin scale. The data suggest that the concentration of PGRN in CSF might be a biomarker for acute NMDAR-AE. Pathological high t-tau levels may indicate a risk for hippocampal sclerosis. The biomarker properties of NfL remain unclear since the levels decrease under treatment, but it could not predict severity of disease in this small cohort. According to our results, we recommend to measure in clinical practice PGRN and t-tau in the CSF of patients with AE.

## Introduction

Since the appearance of antibody-mediated autoimmune encephalopathy (AE) numerous antibodies (ab) have been linked to different clinical symptoms such as limbic encephalitis, faciobrachial dystonic seizures or dementia-like symptoms (1–3). Biomarkers of neurodegeneration mirror certain pathomechanisms of neuronal or axonal loss. The measurement of these biomarkers should bear the potential to provide useful information in everyday clinical life, e.g., to monitor the immunosuppressive therapy in Patients with AE. CSF antibody titres in e.g., contactin-associated-protein-receptor-2 (Caspr2)-AE or Leucin-rich glioma inactivated ptotein-1 (Lgi-1)-AE do not mirror the disease course in a linear way (4, 5). Furthermore, the clinical course in several patients suggests that an antibody titer independent pathomechanism might take place (6–8). The underlying mechanisms causing this dichotomy of clinical symptoms and antibody titer are largely unknown (8). One possible explanation could be the effect of the long survival of plasma cells in the brain (9). The brain-resident plasma cells itself cannot be measured as yet, but the damage possibly caused by autoantibodies should be detected via biomarkers for neuronal and axonal loss such as t-tau, PGRN, and NfL.

Recently, a direct connection between neurodegenerative mechanisms and AE has been detected in AEs mediated by IgLON5 causing an atypical tauopathy (10). Vice versa a correlation between autoimmune diseases and Tar DNA-binding Protein 43 (TDP-43) mediated neurodegeneration in FTD patients has been reported (11). There is also some debate about IgA-NMDAR-Abs and IgM-NMDAR-Abs (3, 12, 13) causing dementia-like symptoms and mimicking neurodegenerative diseases. Histopathological examinations in patients with AE have been focused on the immunological mechanisms triggered and maintained by the antibodies (8) disregarding a systematic research for markers of neurodegeneration so far. Only one case report of a Lgi-1 antibody positive patient presenting some neurodegenerative markers has been reported at autopsy, without witnessing pathological changes in alpha-synuclein, beta-amyloid, or neurofibrillary tangle (14). MRI findings and long-term neuropsychological data also suggest an involvement of the frontal and temporal lobes in the clinical course of the NMDAR-AE and voltage-gated-potassium channel (VGKC)-complex-mediated AE (15–18). Another group has looked at the glial fibrillary acid, NfL and t-tau levels in patients with suspected AE (19). Their group only encompassed four patients with NMDAR-AE and one Lgi-1 patient with most of them having a status epilepticus (SE) before, as SE is known for confounding the protein levels in the CSF (20, 21). They found higher NfL and t-tau levels in all patients, which is most likely due to the SE before. In pediatric opsoclonus-myoclonus syndrome caused by antibodies with intracellular epitopes, immunosuppressive treatment has shown to decrease the CSF-Neurofilament light chain levels together with a concomitant clinical improvement (22, 23).

Here, we examined concentrations of PGRN, NfL, and t-tau, well-established biomarkers of neurodegeneration, in CSF and serum of 38 patients with antibody positive AE. The aim of this study is to investigate if these proteins are possible biomarkers in Patients with AE. Also, the knowledge about the biomarkers of neurodegeneration CSF-levels may give clues about the pathological mechanisms in these patients.

## Methods

### Clinical cohort

This retrospective study was performed according to the local ethical committees in Berlin, Potsdam, Brandenburg, Magdeburg and Bielefeld, respectively. All patients gave written and informed consent (ethics committee approval number 100/16). We included only patients with a proven AE by clinical symptoms as recommended by Graus et al. (24) and detection of pathological antibodies with extracellular epitopes via indirect immunofluorescence tests. The samples of AE-Patients were collected from April 2013 until October 2017 in Berlin, Potsdam, Tübingen, Bielefeld, and Magdeburg where their samples were initially stored at −80°C and sent to Magdeburg. Every sample was stored in Magdeburg at −80°C and all biomarkers were measured in Magdeburg. All samples were run in duplicate with the mean taken as result. Samples were measured over time and not in a batch.

All patients received a lumbar puncture as part of the diagnostical work up when presenting for the first time on the ward and for antibody titre control in follow up depending on each individual disease course. At least 5 ml up to 13 ml CSF was taken and serum was collected in serum separator tubes and centrifuged at site. Every patient (*n* = 38) suffered from a limbic encephalitis including its variants, e.g., limbic encephalitis together with vegetative and/or peripheral neurological symptoms. All patients improved under immunosuppressive therapy. Treatment comprised methylprednisolone (dosage ranges from 3 g up to 18 g during disease course), cyclophosphamid or rituximab (with a minimum dosage of 2g), plasmapheresis or ivIG. We had no patients with a relapse in this cohort. None of the patients had a status epilepticus before lumbar puncture confounding the biomarker levels because of neuronal and axonal death due to the status epilepticus.

Two patients with other antibodies targeting extracellular epitopes [AMPAR and GABAB(R)] were not considered in the statistical analysis but for **Figure 3** to illustrate the biomarker and MRI timeline an AE patients. See Table 1 for the different AE cohorts used per biomarker. We had follow up data in 13 patients. All other patients (samples) were categorized into either initial/acute phase or under treatment.

**Table 1:** Epidemiological data.

**Biomarker**	**Antibody**	**Initial [age/sex]**	**Follow up [age/sex]**
Progranulin	NMDAR	**6** [28.9/6:0]	**17** [27.1/16:1]
	Caspr2	**8** [61.9/2:6]	**5** [67.1/0:5]
	Lgi-1	**7** [69.0/5:2]	**6** [63.2/3:3]
T-Tau	NMDAR	**3** (38.7/3:0]	**3** (38.7/3:0]
	Caspr2	**5** [69.6/0:5]	**5** [69.6/0:5]
	Lgi-1	**3** [63.7/2:1]	**3** [63.7/2:1]
Nfl	NMDAR	**3** (38.7/3:0]*	**13** (30.2/12:0]
VGKC-group{	Caspr2	**6** (68.3/1:5]	**5** [67.1/0:5]
	Lgi-1	**3** [63.7/2:1]	**3** [63.7/2:1]
Control group young	None	**24** [29.9/18:6]	*
Control group old	None	**21** [60.0/9:12]	*
		*not part of statistics	
		sex = female:male	

*Overview of the single groups tested in this study. Number of patients included in bold, sex and mean age are listed*.

#### Magdeburg group

Patients with AE who were identified and treated in Magdeburg (*n* = 13) build a special cohort, because we could e.g., compare serial MRIs to look for AE caused lesions, basic CSF parameters, the outcome with the modified Rankin scale, t-tau, and other biomarker levels in the CSF and ab titer. The modified Rankin scale in this cohort was assessed by two experienced neurologist (PK, DB). Six out of thirteen had a paraneoplastic origin and one patient a postinfectious origin of the AE.

#### CSF-neurofilament light chain measurements

**Figure 1:**
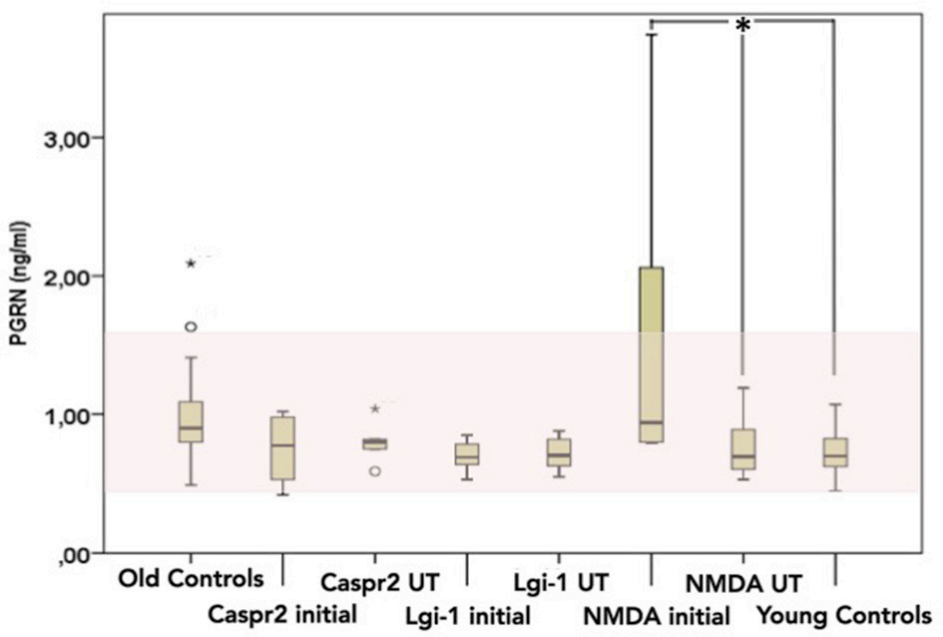
CSF Progranulin levels [pg/ml] in patients with AE (*n* = 36) measured divided by antibody and by phase of disease (initial vs. under treatment). Significant results are marked with a *. Normal range is marked in light red. Line represents the median and the error bars represent the interquartile range. Black points are outliers.

We divided the AE cohort into three groups regarding the CSF-NfL measurements in order to be able to perform a sufficient statistical analysis (see Table 1). One group with voltage-gated potassium channels (VGKC) mediated AE (comprising the Caspr2 and Lgi-1 patients) subdivided in an “initial” (*n* = 9) and “under treatment,” meaning after several immunosuppressive therapies, subgroup (*n* = 8) and one group with NMDA patients under treatment (*n* = 13) (see Table 1 and Figure 1). There were not enough NMDA patients who would fit into an initial/acute phase group (*n* = 3). Therefore, this group could unfortunately not be part of the statistical analysis.

Neurofilament light chain was measured with a commercial ELISA (Umandiagnostics, Sweden, catalogue number 10-7001 CE). The sensitivity of this assay is 31 pg/ml. The cut-off for pathological levels was set at 3523 ng/ml (mean (1823[ng/ml]) + 2 standard deviation (850[ng/ml]) above). Intraessay coefficient of variance is 7.4% and interessay coefficient of variance is 6%. NfL is a stable protein, which can be measured in the CSF even though the sample was on room temperature for up to 8 days (25). Therefore, we could measure NfL in samples not collected at Magdeburg, We used an already established control group at Magdeburg (*n* = 34, mean age = 64.4, CSF-NfL = 1823 ± 850 [ng/ml]) comprising patients with other than neuroinflammatory or neurodegenerative diseases (e.g., headache, suspicion of infection in the CNS etc.).

#### Total-tau measurements

The correct measurement of t-tau due to manufacturer's instructions requires different than standard processing of the CSF samples to create a cell-free sample excluding this parameter from a retrospective study. We yielded t-tau levels only in the Magdeburg group, as t-tau is part of the routine in Magdeburg but not in the other centers involved. Total-Tau levels were determined using a commercially available single-parameter ELISA kit [Innogenetics, Ghent, Belgium, catalogue numbers: 81572 (962-CE) and 81573] established in our routine diagnostical work up. Intraessay coefficient of variance is 13.2% and interessay coefficient of variance is 11.5%. The pathological levels were considered according to the manufacture guidelines.

#### CSF-progranulin measurements

**Figure 2:**
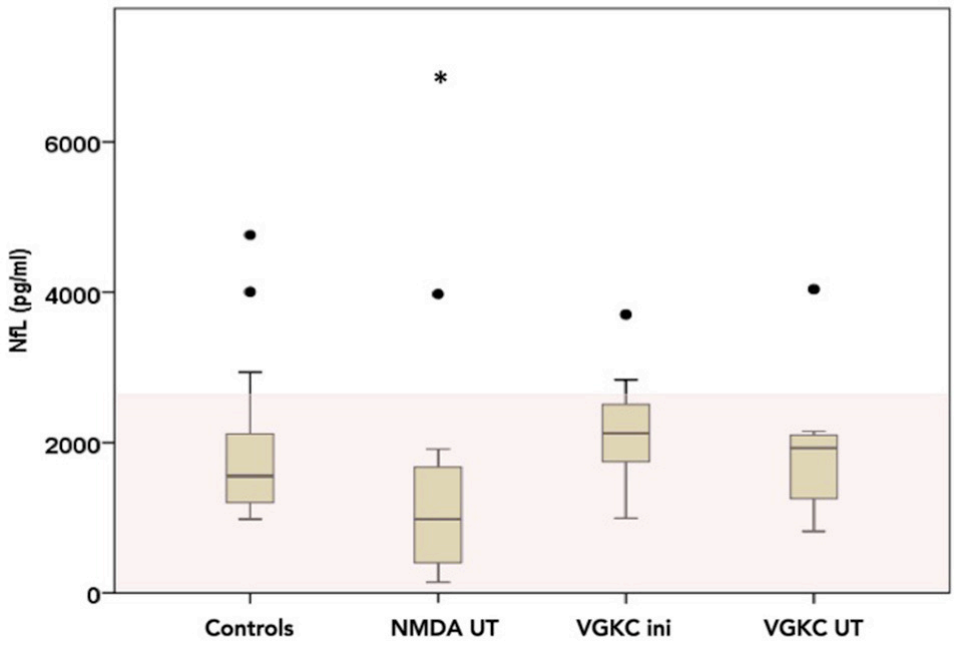
CSF-Neurofilament light chain (NfL) levels divided into three possible groups (NMDA under treatment *n* = 14, VGKC initial *n* = 7 and under treatment *n* = 6) as box plots (number of measurements, *n* = 30). The area in light red marks the normal range. Please note that all levels are inside the normal range. Line represents the median and the error bars represent the interquartile range. White points mean outlier and star extreme outliers.

We measured PGRN in CSF and serum of 36 Patients with AE. We divided our AE cohort (*n* = 36) into three groups according to the antibody causing the limbic encephalitis when looking statistically at the CSF-PGRN levels: one Lgi-1 group (*n* = 10, mean age = 69.2), one Caspr2 group (*n* = 8, mean age = 61.9) and a NMDAR group (*n* = 18, mean age = 27.1). These three groups were subdivided into two subgroups respectively one before and one after initiating immunosuppressive therapy (again, called “initial” or “under treatment”) (see Table 1 and Figure 2).

A commercial ELISA was performed to determine the levels of PGRN (Human Progranulin ELISA Kit, Mediagnost, Reutlingen, Germany, catalogue number E103) according to the manufacturer's instructions. Intraessay coefficient of variance is 4.4% and interessay coefficient of variance is 8.0%.

We established two control groups for PGRN measurements. Since PRGN levels are age dependent, we build a younger control group (*n* = 24; mean age 29.3 years; 18–40yrs) and one older group (*n* = 39; mean age 66.3 years, 50–75 years) and correlated it to age. The patients from these control groups had other neurological diseases than neuroinflammatory or neurodegenerative (e.g., acute headache, excluding neuroinflammatory diseases, no epilepsy patients). CSF-PGRN level was considered pathological, when the CSF-PGRN levels per control group were two standard deviation above or below the mean for each control group respectively.

#### Antibody detection

Antibody detection was either performed at the antibody laboratory Bielefeld, at the University Hospital Schleswig-Holstein, Department of Neuroimmunology Kiel and Lübeck or at the Institute of Molecular and Clinical Immunology Magdeburg. Standard indirect immunofluorescence tests were performed on antigen-specific transfected Hek293 cells as commercially available and used in clinical routine (EUROIMMUN, Lübeck, Germany, catalogue numbers: FA 112d-1003-6, FA 112d-1003-51, FA 1430-1003-1) for each patient revealing the specific antibody and the titer in CSF and serum.

### Statistics

Statistics were calculated by SPSS 21.0 (IBM, Seattle, USA). Since group size and variances were not equal non-parametric tests were applied. For comparison of more than two different groups Kruskal-Wallis test was used with tamhanes *post-hoc* analysis. Group comparison of two groups was analyzed with Mann-Whitney-U test when they were independent and with the Wilcoxon test when the samples were paired to each other. For correlational analysis of serum and CSF PGRN Spearmann-rho correlation was applied. Tests were considered significant when reaching *p* < 0.05. There was no test for outliers applied.

## Results

### Cohorts

Since the CSF-PGRN level is age-dependent we established a younger control group aged 18–40 years (*n* = 24, CSF-PGRN = 0.72 ± 0.17 ng/ml, Serum-PGRN = 36.4 ng/ml, mean age = 29.3 years) and a control group 50-75 years (*n* = 39, CSF-PGRN = 0.94± 0.22 ng/ml, Serum-PGRN = 28.5 ng/ml mean age = 66.3 ± 9.8 years). Spearman correlation statistics revealed a significant correlation between CSF-PGRN and age (*r* = 0.275, *p* = 0.02).

**Figure 3:**
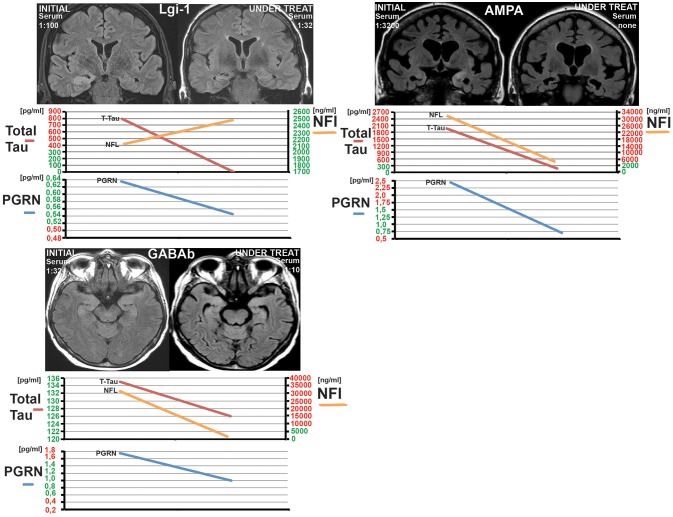
Three examples of patients from the Magdeburg cohort (patient 5, 12, and 13 see Table 1) showing the under treatment MRI together with the CSF-Neurofilament light chain (NfL), CSF-Progranulin (PGRN), and Total tau. Normal levels are marked in green at the y-axis and pathological levels are marked in red. Notice the hippocampal sclerosis in the Lgi-1 patient and the complete recovery from the oedema in the GABA(B)R-AE-patient without a clear sign of a hippocampal atrophy. The third patient has a AMPAR-AE. The follow up in this patient shows a severe bilateral hippocampal atrophy. Nevertheless the patient improved over years.

In the Magdeburg group 9/13 had a FLAIR-intense signal in the limbic system on the MRI. Five out of thirteen patients developed a hippocampal sclerosis due to AE (see Table 2 and Figure 3). Every patient with pathologically elevated t-tau levels developed a hippocampal sclerosis. The one patient, who developed a hippocampal sclerosis without elevated t-tau levels but elevated NfL concentrations, was administered after he was already treated and had a hippocampal sclerosis. Therefore the CSF was taken and measured ~8 months after beginning of the AE and treatment. On the contrary only 4 out of 7 patients with pathological NfL levels developed a hippocampal sclerosis. Every marker of neurodegeneration and the modified Rankin scale (mRS) decreased after initializing the immunosuppressive treatment paralleled by a decrease in antibody titre (see Figure 3 for examples and Table 2 for the follow up data).

**Table 2:** Biomarkers in the Magdeburg cohort.

	**Antibody**	**Titer**	**Age**	**Year**	**T-tau**	**NFlight**	**CSF-PGRN**	**Serum-PGRN**	**Cell count**	**mRS**	**Comment**
		**CSF/Serum**	**Onset**		**pg/ml**	**ng/ml**	**pg/ml**	**pg/ml**	**CSF**		
					>370	>3488	< 0.54–1.4>	< 18–54>	< 4 cells/mm2		
Patient 1	Caspr2	1:128/1:32000	60–65	2013	253	12342	0.76	n.a.	3	5	Temporal FLAIR-intense signal
		none/1:375		2015	133	2152	0.8	32.5	0	3	Paraneoplastic origin
		none /1:128		2015	108	2055	0.61	30.62	1	3	Hippocampal sclerosis
Patient 2	Caspr2	1:64000/1:750000	75–80	2014 #	292	2047	1.02	27.11	13	5	Temporal FLAIR-intense signal
		1:6000/1:96000		2015	213	2123	0.87	30.63	17	1	
Patient 3	Caspr2	1:320/1:3200	70–75	2015 #	367	4536	0.84	21.94	0	1	Normal MRI
		1:320/1:3200		2015 #	328	3580	0.74	22.05	3	1	
Patient 4	Caspr2	1:3200/1:1000	70–75	2016 #	314	3705	0.95	35.18	6	5	Temporal FLAIR-intense signal
		1:10 /1:2000		2016	317	4039	0.82	51.57	2	1	
Patient 5	Caspr2	1:320/1:4000	66–70	2017 #	349	2159	1.01	32.04	5	4	Normal MRI
		1:8/1:1000		2017	375	2586	0.82	43.58	2	0	
Patient 6	Lgi-1	none/1:100	60–65	2015 #	796	2128	0.63	32.97	1	3	Temporal FLAIR-intense signal
		none/1:32		2015	>11	2493	0.54	38.92	0	0	Hippocampal sclerosis
Patient 7	Lg-1	1:2/1:1000	65–70	2017 #	128	993	0.74	19.97	1	3	Paraneoplastic origin
		none/1:320		2017	149	1273	0.73	23	0	0	
Patient 8	Lgi-1	1:2 /1:160	60–65	2017 #	197	1582	0.65	27.33	0	3	Temporal FLAIR-intense signal
		1:20/1:10		2017	161	1233	0.68	30.12	2	0	
Patient 9	NMDA	1:32/1:320	25–30	2010 #	141	n.a.	0.76	29.11	1	5	Paraneoplastic origin
		1:10/1:100		2014	105	390	0.89	33.06	3	1	
		1:1/none		2015	58	553	1.3	n.a.	2	1	
Patient 10	NMDA	1:40/1:80	25–30	2016 #	869	38650	1	23.08	7	5	Temporal FLAIR-intense signal
		1:10/1:5		2016	229	20736	0.71	17.86	6	2	Hippocampal sclerosis
		1:5/1:5		2017	68	6841	0.71	25.43	3	1	Postinfectious origin
Patient 11	NMDA	none/1:10	60–65	2014 #	801	28791	1.53	35.51	96	4	Hippocampal sclerosis
		none/1:10		2014	372	42286	1.19	32.9	43	0	Temporal FLAIR-intense signal
		none/none		2015	192	3975	0.96	35.4	9	0	Paraneoplastic
Patient 12	GABA(B)R	1:320/1:32	50–55	2016 #	135	32029	1.79	37.21	47	5	Paraneoplastic origin
		1:320/1:1		2016	168	21439	1.03	37.5	1	3	Temporal FLAIR-intense signal
		none/1:10		2016	126	3581	0.95	n.a.	1	2	
Patient 13	AMPA	1:32/1:3200	70–75	2014 #	1950	32151	2.47	34.21	43	5	Paraneoplastic origin
		1:8/1:375		2014	1984	20354	1.23	38.22	90	4	Temporal FLAIR-intense signal
		1:1/none		2015	173	2892	0.7	n.a.	3	2	Hippocampal sclerosis
		#	Before treatment								
		n.a.	Not available								

*Complete list of all 13 patients in the cohort where follow up data is available including antibody, antibody serum titer, age, year at which the sample has been obtained, Neurofilament light chain (NfL), Total-tau (T-Tau), CSF-Progranulin (PGRN), Serum-Progranulin, cell count, modified Rankin scale (mRS) and comments on MRI abnormalities and putative pathogenesis; n.a., not available; #, timepoint before start of the immunosuppression*.

### Neurofilament light chain

NfL was pathologically high (>3523 pg/ml) in 7/23 patients at different stages of the AE (see Figures 2, 3 and Table 2). Out of these seven patients, four had a paraneoplastic origin of the AE and one a postinfectious origin. Furthermore, 5/7 patients had a FLAIR-intense signal in the limbic areas on the MRI, which normalized during immunosuppressive treatment. This decrease in FLAIR signal was mirrored by a decrease in NfL-levels reaching normal NfL levels during disease course (see Figure 3 and Table 2). Three out of five patients who developed hippocampal sclerosis had elevated CSF-NfL levels additionally to the also elevated t-tau. Solely elevated CSF-NfL was found in 4 patients. We correlated the leukocyte count to the NFL levels in the Magdeburg cohort and found no correlation (Spearmans *r* = 0.625).

There was a trend toward a lower NfL in the NMDA under treatment group (CSF-NfL = 1455 [pg/ml], range 142–6841[pg/ml]) compared to the VGKC under treatment group (CSF-NfL = 2164 [pg/ml], range 821–4039 [pg/ml]) (Mann-Whitney U test *p* = 0.052 Z = −1.941), while there was no difference comparing the VGKC subgroups initial vs. under treatment (Wilcoxon test *p* = 0.735 and Z = −0.338).

### Total tau

Looking at the initial t-tau in our patients (before initiating the treatment) revealed a pathologically high t-tau in 4 patients (see Table 2). All 4 patients had MRI-FLAIR intense signals in the temporal lobe/limbic system and subsequently a hippocampal sclerosis. Immunosuppressive treatment did show an effect on the modified Rankin scale and also resulted in a decrease of t-tau in these patients. The FLAIR signal also decreased in these patients (see Figure 3). In the other 8 patients without elevated t-tau levels immunosuppressive therapy had no effect on t-tau levels. A concomitant tumor had no impact at all on the t-tau levels. (see Table 2).

### Progranulin

The mean CSF-PGRN levels were pathologically high in the initial NMDAR-group (CSF-PGRN = 1.55 ± 1.1 ng/ml) reaching significance when compared to the NMDAR under treatment group (Mann-Whitney-U test Z = −2.5 and *p* = 0.012) and also when compared to the age-matched healthy group (Mann-Whitney-U test Z = −2.689 and *p* = 0.007). Serum PGRN levels were inside normal ranges in every AE patient and did not change after immunosuppression (see Figure S1). CSF-PGRN and CSF-Serum ratios ranged from 0.01 to 0.1, still CSF-PGRN is show to originate from the CNS when measured in the CSF (26, 27) We also could not find a correlation between Serum-PGRN and CSF-PGRN in all groups (Spearman-rho coefficient *r* = 0.17, *p* = 0.3) (Figure S1 in the Supplement).After initiating the immunosuppressive therapy CSF-PGRN dropped to normal levels (CSF-PGRN = 0.75 ± 0.2 ng/ml) in the “under treatment”-group.

Mean CSF-PGRN levels were normal in the Lgi-1 “initial” group (CSF-PGRN = 0.71 ± 0.11 ng/ml) and in the under treatment group (CSF-PGRN = 0.72 ± 0.12 ng/ml) (see Figure 1). Comparing these results to the age-matched group revealed significant differences (Kruskal-Wallis test: for “initial” PGRN: *p* = 0.009, X^2^ = 9.4, for follow-up: *p* = 0.04, X^2^ = 6.3). There were significantly lower levels in tamhane *post-hoc* in the Patients with AE (Lgi-1 initial vs. control *p* = 0.009 and *p* = 0.041 for the under treatment vs. control) The CSF-PGRN levels in both Caspr2 groups were inside the normal range (mean CSF-PRGN initially = 0.75 ± 0.23 ng/ml and mean CSF-PGRN under treatment = 0.8 ± 0.16 ng/ml) without significant results when compared to the age-matched controls (Caspr2 initial vs. under treatment *p* = 0.35 and Caspr2 under treatment vs. control *p* = 0.92). There was no difference between the “initial” and the “under treatment” group in the Lgi-1 and Caspr2 cohorts, respectively.

### Follow up data

In summary, in all cases with an elevated biomarker of neurodegeneration in the CSF a decrease of biomarkers, ab titres, and mRS was observed following immunosuppressive treatment in all patients (see Table 1 and Figure 3) regardless if the origin was postinfectious, paraneoplastic, or cryptogenic. There were two patients with all biomarkers simultaneously elevated. None of these parameters could predict a hippocampal sclerosis for sure on the one hand; on the other hand every patient who developed a sclerosis had either elevated t-tau or NfL levels with t-tau appearing to be more predictive.

## Discussion

We could show for the first time that biomarkers of neurodegeneration originating from CNS are mirroring the clinical and probably neuroimmunological course of patients suffering from AE associated with antibodies to extracellular epitopes. CSF-PGRN is elevated in patients with NMDAR-AE during the acute phase. Furthermore, biomarkers of neurodegeneration such as t-tau together with CSF-NfL in Patients with AE might be predictive of the clinical outcome especially for developing a hippocampal sclerosis. The pathologically elevated biomarkers correlated with the mRS, the clinical course and the antibody titre. Besides Progranulin in acute NMDAR-AE, NfL, PGRN and t-tau did not seem to be restricted to one special autoantibody mediated AE. This may be due to the fact that the neuronal and axonal damages in general are mirrored and not the distinct pathomechanisms of each putative pathological autoantibody.

### Neurofilament light chain

NfL has been proven as an excellent marker of axonal loss (28). It seems very unlikely and there has been no data on whether peripheral tumors such as teratomas nor other neuroendocrine tumors can influence the CSF-NfL levels as possible confounders in our study. The co-occurrence of MRI changes, hippocampal sclerosis, and elevated NfL levels in our Magdeburg cohort is pointing at a pathomechanism causing the edema and subsequently the FLAIR signal resulting in the axonal dysfunction and subsequently increased NfL levels in the CSF. Other than in neurodegenerative diseases such as FTD (29, 30) the axonal loss in AE ceases after initiating sufficient immunosuppressive therapy as seen by the group of Pranzatelli in pediatric patients with opsoclonus-myoclonus syndrome caused by antibodies with intracellular epitopes (22, 23). Constantinescu et al. also measured CSF-NfL in four Patients with NMDAR-AE with AE and one Lgi-1 patient (19). Three NMDA-patients had a status epilepticus and highly pathological CSF-NFL levels, which has been seen in SE for nearly every marker of neuronal death (20, 21). In our cohort none had a status epilepticus confounding the biomarkers. This is possibly the reason why we found normal CSF-NfL levels in all measured Patients with NMDAR-AE except for the one with postinfectious origin. The meningoencephalitis with subsequent neuronal and axonal loss before the AE might be a reason for the elevated NfL levels in this patient since the infection was only 7 weeks apart from the AE. Our results are also much more in line with the known pathomechanism in NMDAR- AE (31, 32) where only marginal neuronal damage occurs and the main reason for the clinical symptoms is most likely the internalization of the NMDA-receptor. The one Lgi-1 patient (without SE) in the cohort of Constantiescu et al had normal NfL levels as our entire Lgi-1 group.

Total-Tau is a better marker for neuronal death as NfL (see below). However, FLAIR intense signals in the hippocampus as a consequence of disturbances of neuronal membrane function did correlate with NfL levels in our small cohort.

### Total-tau

Total-tau is an excellent marker for neuronal death (21, 33) 4/5 patients who developed a hippocampal sclerosis had pathological elevated t-tau levels in our Magdeburg cohort (*n* = 13). This is well in line with the current concept of the pathomechanisms leading to a sclerosis (34). Although, patient 1 (see Table 2) with the Caspr2 AE who was already treated months before admission to Magdeburg had only elevated CSF-NfL and normal t-tau levels and a hippocampal sclerosis (see Table 2). Sadly, it was not possible to measure NfL and t-tau levels in this patients initial CSF.

In sum, the measurement of t-tau may be a good marker before treatment decision in suspected autoimmune encephalitis or before deciding on the further immunosuppressive treatment but is limited to laboratories with expertise in measuring t-tau.

### Progranulin

PGRN is playing a role in autoimmune mediated diseases such as rheuma or bowel disease or status epilepticus or in suppression of neuroinflammation (30, 35–37) Recently, EpiphrinA2 as a part of the Ephrin receptor kinase has been identified as functional receptor of PGRN and the potential of PGRN phosphorylating and activating the EpiphrinB2 receptor (38) linking it to the dysfunction in the EpiphrinB2 pathway known in AE mediated by autoantibodies against the NMDA-receptor (39, 40). The distribution in the fronto-temporal structures (41), the possible common link with the AE mediated by NMDAR via the EpiphrinA2-EpiphrinB2 pathway and the known role as a mediator in neuroinflammation and autoimmunity makes PGRN an interesting protein in AE.

We detected elevated CSF-PGRN levels in our NMDA-patients with a severe ongoing AE. On the other hand, CSF-PGRN was low in patients suffering from Lgi-1-AE sometimes reaching levels of FTD patients (29) in contrast to Patients with NMDAR-AE and controls. The significance of this low CSF-PGRN is doubtful because none of the CSF-PGRN levels normalized after initiating the immunosuppressive therapy. Also most CSF-PGRN levels in the patients with Lgi-1-AE were still inside the normal range.

When looking at the t-cell or b-cell specific cytokine patterns in the patient's CSF suffering from NMDAR-AE several groups have seen a massive b-cell predominant cytokine pattern in the beginning especially in CXCL-13 levels (42, 43) and then a decrease in follow up. Also, cytokine pattern associated with t-cell activation were detectable throughout the course of AE without relevant changes. This course in cytokine levels could explain the elevated Progranulin levels in patients having acute NMDAR-AE.

PGRN in Serum and CSF was not elevated in patients with AE due to paraneoplastic origin although PGRN is also known as a tumor marker for certain tumors such as Lymphomas (44) The missing correlation between the serum-PGRN and CSF-PGRN is pointing at a cerebral origin of the CSF-PGRN as already seen in other diseases (26, 27). Overall, this result is probably due to an affection of the CSF-PGRN pathway in acute NMDAR-AE but needs more *in vivo* and *in vitro* experiments to be further examined.

One major limitation of the study is the small sample size in every subgroup tested. This fact is due to the very low numbers of patients with AE overall. Although total numbers are too small to draw a final conclusion the t-tau levels together with the CSF-NFL levels seem to best characterize the stage of neuronal death in the brain. The diagnostic value of NFL levels in the CSF should be evaluated in further studies. Another limitation is that we only had follow up data in 13 patients limiting our knowledge about MRI, mRS, and ab titres. Another limitation of the study is that due to the scarcity of the diseases measurements of the biomarkers could not be done in a batch but on demand.

A larger study should be conducted to further elucidate the correlation of these interesting parameters how they could contribute to therapeutic decisions.

## Conclusion

NfL, t-tau and PGRN could be potential biomarkers of neuronal or axonal loss in patients suffering from AE. Especially, the Patients with NMDAR-AE have elevated PGRN levels at the acute phase of the AE. This fact further strengthens the hypothesis of a pathological change in the Epiphrin receptor metabolism in NMDAR patients. CSF-PGRN may be a marker for acute NMDA-AE.

Furthermore, we strongly recommend measuring NfL and t-tau in the CSF of every patient with AE although one biomarker for itself could not predict all hippocampal sclerosis in this pilot study. Pathological levels of a biomarker of neurodegeneration should be considered as an on-going AE and may be taken into account when planning further therapy.

## Ethics statement

This study was carried out in accordance with the recommendations of ethics committee of the University hospital Magdeburg (number 100/16). The protocol was approved by the ethics committee of the University hospital Magdeburg. All subjects gave written informed consent in accordance with the Declaration of Helsinki.

## Author contributions

PK has access to all the data and takes responsibility for the data, accuracy of the data analysis, and the conduct of the research design or conceptualization of the study and analysis or interpretation of the data and drafting or revising the manuscript for intellectual content; HP and DB: Design and conceptualization of the study; analysis or interpretation of the data; drafting and revising the manuscript for intellectual content; LT: Conceptualization of the study; analysis of the data; drafting the manuscript for intellectual content; DV-W, JS-A, and SS: Conceptualization of the study; drafting the manuscript for intellectual content; WM, DR and FL: Conceptualization of the study; analysis and interpretation of the data; drafting and revising the manuscript for intellectual content; H-JH: Design of the study; drafting the manuscript for intellectual content.

### Conflict of interest statement

PK has received consulting fees from Eisai (Germany). JS-A obtained honoraria for speaking engagements from Boehringer Ingelheim (Germany) and Bristol-Myers Squibb (Germany). LT: Saarland University, LT and others filed 61/730,772 which covers means and methods for detecting autoimmune disorders in which progranulin antibodies may be involved. FL runs an antibody detection laboratory in Kiel, Germany where part of the work has been performed. The remaining authors declare that the research was conducted in the absence of any commercial or financial relationships that could be construed as a potential conflict of interest.

## Abbreviations

Ab: Antibody
AD: Alzheimer's disease
AE: Autoimmune mediated Encephalitis
AMPAR: alpha-amino-3-hydroxy-5-methyl-4-isoxazolepropionic acid receptor
Caspr2: Contactin associated protein 2
CSF: cerebrospinal fluid
FTD: Frontotemporal dementia
GABAB(R): Gabaaminobutyrat-B subunit receptor
Lgi1: Leucin-rich glioma inactivated protein1
NMDAR: N-methyl-D-aspartate receptor
NfL: Neurofilament light chain
PGRN: Progranulin
SE: Status Epilepticus
t-tau: T-tau
VGKC: Voltage-gated potassium channel.
