# Supplementary figures and images for: Biomarkers of Neurodegeneration in Autoimmune-Mediated Encephalitis
Source: Front Neurol. 2018 Sep 19;9:668. doi: 10.3389/fneur.2018.00668 (PMC6156245; doi:10.3389/fneur.2018.00668)

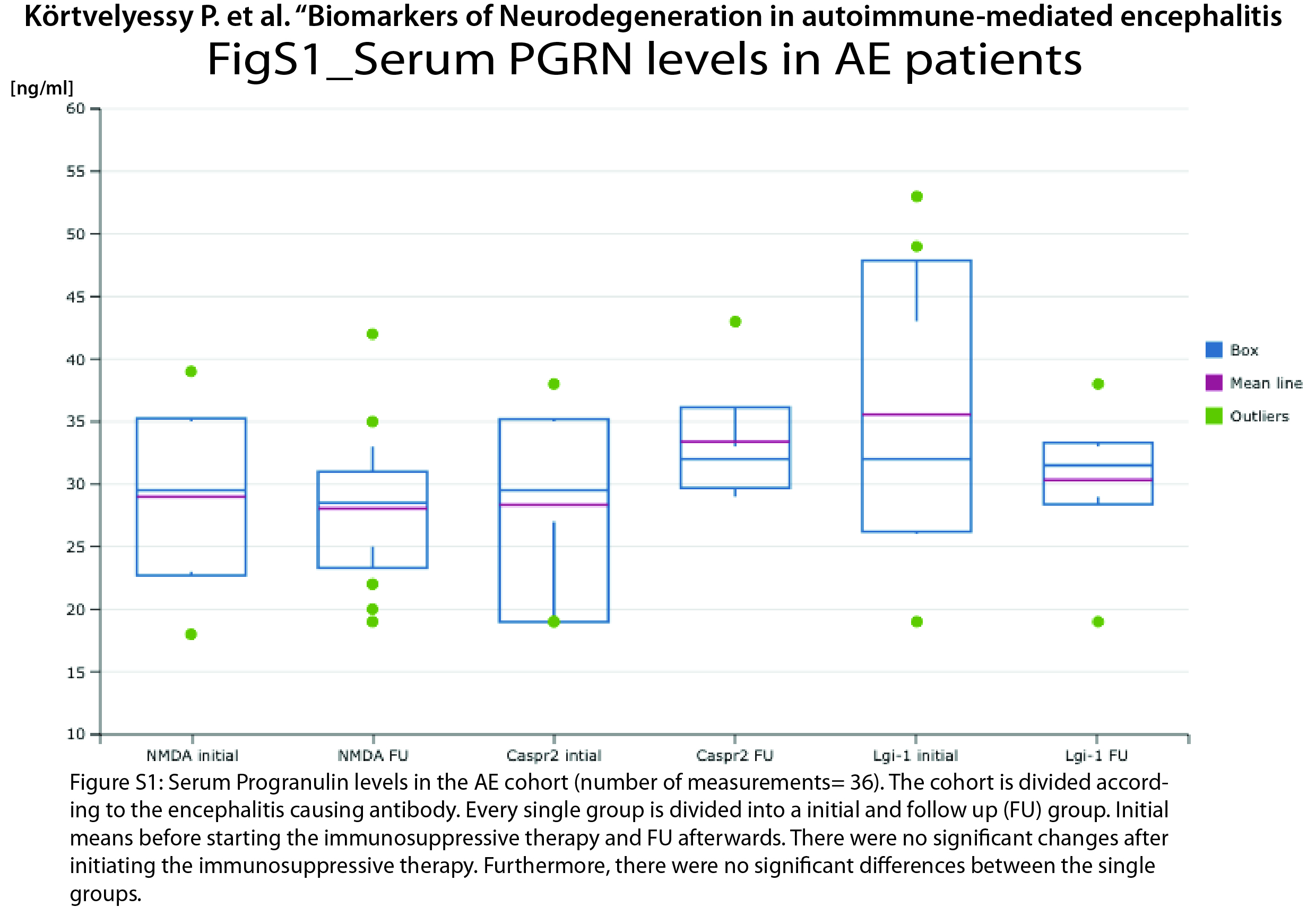

Supplement: Supplementary file 1 [file Image_1.JPEG]
